# HPLC and GC-MS Determination of Bioactive Compounds in Microwave Obtained Extracts of Three Varieties of *Labisia pumila* Benth

**DOI:** 10.3390/molecules16086791

**Published:** 2011-08-09

**Authors:** Ehsan Karimi, Hawa Z.E. Jaafar

**Affiliations:** Department of Crop Science, Faculty of Agriculture, University Putra Malaysia, UPM Serdang, Selangor 43400, Malaysia; Email: Ehsan_b_karimi@yahoo.com

**Keywords:** microwave assisted extraction, antioxidant properties, phytochemicals composition, RP-HPLC analysis, GC-MS screening

## Abstract

Microwave extraction of phytochemicals from medicinal plant materials has generated tremendous research interest and shown great potential. This research highlights the importance of microwave extraction in the analysis of flavonoids, isoflavonoid and phenolics and the antioxidant properties of extracts from three varieties of the Malaysian medicinal herb, *Labisia pumila* Benth. High and fast extraction performance ability, equal or higher extraction efficiencies than other methods, and the need for small samples and reagent volumes are some of the attractive features of this new promising microwave assisted extraction (MAE) technique. The aims of the present research were to determine the foliar phenolics and flavonoids contents of extracts of three varieties of *L. pumila* obtained by a microwave extraction method while flavonoid, isoflavonoid and phenolic compounds were analyzed using RP-HPLC. Furthermore, the antioxidant activities were measured by the DPPH and FRAP methods and finally, the chemical composition of the crude methanolic extracts of the leaves of all three varieties were analyzed by GS-MS.

## 1. Introduction

Antioxidants are chemical compounds extremely useful to humans. They have ability to reduce free radicals and/or to decrease their rate of production and lipid peroxidation in human bodies that cause various human diseases and aging [1]. In general, there are two basic categories of antioxidants, the synthetic and the natural ones. Plants provide us with rich sources of natural antioxidants [2]. Phytochemicals such as phenolic and flavonoid compounds commonly found in plants act as antioxidants with redox and metal chelating properties. Furthermore, other biological activities like anti-inflammatory, antidiarrheal, antiulcer, antiviral, antiallergic and vasodilatory actions have also been reported for these compounds [3].

Extraction is the main step for the recovery and isolation of bioactive phytochemicals from plant materials, before component analysis. The analysis and extraction of plant matrices play an important role in the development, modernization and quality control of herbal formulations. The microwave extraction process has many advantages such as increasing the yield in shorter periods, and at the same time using less solvent. When choosing parameters for this extraction method, some of the important considerations are the physical parameters, including solubility, dielectric constant, and the energy dissipation factor. 

Microwave energy is a non-ionizing radiation that results in molecular motion by migration of ions and rotation of dipoles [4]. Due to economic and practical aspects, much attention has been paid to microwave extraction compared to traditional methods for the extraction of metabolites from plants [5]. Many bioactive compounds can be extracted with microwave-assisted extraction such as secondary metabolites like flavonoids and phenolics [4], essential oils [6], saponins [7] and synthetic estrogenic steroids [8]. Most higher plants have been used in traditional medicine for a long time and about 13,000 plant species have been used as drugs throughout the World [9]. Plant secondary metabolites are important sources of various fine chemicals (phytochemicals) that are used directly or as intermediates for the production of pharmaceuticals.

*Labisia pumila* Benth. (*Myrsinaceae* family) locally known in Malaysia as Kacip Fatimah is a woody, small sub herbaceous plant with creeping stems [10]. Stone [11] had categorized three varieties of this herb in Malaysia, namely *L. pumila* var. *alata*, *L. pumila* var. *pumila* and *L. pumila* var. *lanceolata*. Each of these varieties has different uses. Recently, it was reported that the bioactive compounds of *L. pumila* consisted mainly of resorcinols, flavonoids and phenolic acids [12,13,14,15,16]. These compounds can be enriched by micro-climatic manipulation [13,14], and have been implicated as natural antioxidants, which can safely interact with free radicals and terminate their chain reactions before vital molecules could be damaged. The use of a microwave extraction method for the determination of the polyphenolics content in the three varieties of *Labisia pumila* plants has not been reported before. Hence, this research was performed to investigate the accumulation of bioactive compounds such as flavonoids, isoflavonoids and phenolics in the leaves of three varieties of *L. pumila*, and their antioxidative effects using extracts obtained by microwave-assisted extraction. The chemical compositions of bioactive compounds of these varieties were also investigated by GC-MS screening.

## 2. Results and Discussion

### 2.1. Total Phenolics and Flavonoids Contents

Phenolic and flavonoid compounds, as important phytochemicals, are present in vegetables, fruits and cereal grains. These secondary metabolites are natural antioxidants that have multiple biological effects and play an important role in the defense against cardiovascular disease, aging and cancer [17]. The total phenolics and flavonoids contents of leaves from the three varieties of *Labisia pumila* Benth. extracts are presented in Table 1. The results indicate that *L. pumila* var. *pumila* had a higher total flavonoids content (2.77 mg rutin equivalent/g DW) than var. *alata* (2.49 mg rutin equivalent/g DW) and var*. lanceolata* (2.29 mg rutin equivalent/g DW), but the leaves of var. *alata* contained higher total phenolics (3.48 mg gallic acid equivalent/g DW) than var. *pumila* (3.37 mg gallic acid equivalent/g DW) and var. *lanceolata* (3.23 mg gallic acid equivalent/g DW). The HPLC analysis results also indicated that *L. pumila* var. *pumila* contained various types of flavonoids such as quercetin and daidzein not seen in *L. pumila* var. *alata*, while the phenolic pyrogallol was detected only in *L. pumila* var. *alata*.

**Table 1:** Total phenolics and flavonoids content of the leaves of three varieties of *Labisa pumila* Benth.

Variety	Phenolic Content ^1^	Flavonoid Content ^2^
Alata	3.48 ± 0.01 ^a^	2.49 ± 0.13 ^b^
Pumila	3.37 ± 0.04 ^b^	2.77 ± 0.01 ^a^
Lanceolata	3.23 ± 0.02 ^c^	2.29 ± 0.02 ^c^

**^1^** mg gallic acid equivalent/g DW; **^2^** mg rutin equivalent/g DW; Results are means of three replicates ± standard deviations. Means with the different letters are significantly different from each other at p < 0.05.

Total phenolics and flavonoids content of leaves in all varieties were significantly different from each other. An increase of total phenolic content in some plants upon heating could be due to the cleavage of esterified and glycosylated compounds [18]. Guihua *et al.* [19] also found that the heating process increased the phenolics content due to the cleavage of bound (esterified and glycosylated) forms, thus leading to an increase in free forms.

### 2.2. Antioxidant Assay of Leaves of Three Varieties L. pumila Extracts

Two assays which were the 1,1-diphenylpicrylhydrazyl (DPPH) free radical scavenging activity and ferric-reducing antioxidant power (FRAP) were used to evaluate the antioxidant activity of the extracts. The DPPH assay is based on the color reduction of methanolic DPPH, which was followed by monitoring the decrease in the sample absorbance; a higher color reduction indicates higher anti-radical activity. The FRAP assay depends on the reduction of Fe^+3^ to Fe^+2^ by the samples. The Fe^+2^ formed can be monitored by measuring the formation of Perl’s Prussian blue at 700 nm. The results of the DPPH scavenging assay of the leaf parts of three varieties of *L. pumila* at a concentration of 400 μg/mL are shown in Table 2. The results showed that *L. pumila* var*. alata*, with a value of 65.38 ± 0.15, had higher antioxidative activity, compared to var. *pumila* and var. *lanceolata* (63.83 ± 2.34 and 57.69 ± 1.01, respectively). However, these values were lower than those of the tested antioxidant standards, BHT (butylated hydroxytoluene) and α-tocopherol (99.24 ± 0.25 and 99.73 ± 0.19, respectively).

**Table 2:** DPPH scavenging activities of the leaf part in all varieties of *L. pumila* at concentration of 400 μg/mL. BHT and α-tocopherol were used as positive controls.

Inhibition (%)		
**Variety**	Alata	65.38 ± 0.15 ^b^
	Pumila	63.83 ± 2.34 ^c^
	Lanceolata	57.69 ± 1.01 ^d^
**Control**	BHT	99.24 ± 0.25 ^a^
	α-tocopherol	99.73 ± 0.19 ^a^

All analyses were mean of triplicate measurements ± standard deviation. Results expressed in percent of free radical inhibition. Means with different letters are significantly different from each other at p < 0.05.

Table 3 presents the IC_50_ values (concentration required to inhibit 50% of DPPH radicals) of methanolic extract of the leaf parts in all varieties of *L. pumila*, BHT and α-tocopherol on free radical scavenging activity. IC_50_ values of the leaf parts of the three varieties of *L. pumila* Benth. were lower than the corresponding values of the controls, which were 36.03 and 78.75 µg/mL for α-tocopherol and BHT, respectively. Like the DPPH results, the reductive potential of *L. pumila* Benth. in all three varieties increased in a dose dependent manner. The three varieties of *L. pumila* all appeared to be active in the reduction of Fe^3+^, indicating their antioxidant activity. The reductive potential of *Labisia pumila* extracts in all three varieties and the standards at a concentration of 400 µg/mL (Table 4) were found to be in the descending order of vitamin C > BHT > α-tocopherol > *L. pumila* var. *alata* > *L. pumila* var. *pumila* > *L. pumila* var. *lanceolata* with respective values of 99.59%, 99.18%, 96.17%, 54.84%, 53.11% and 52.17%. 

Many phenolics and flavonoids compounds have been reported to possess potent antioxidant activity and anti-cancer, anti-carcinogenic, anti-bacterial, anti-viral or anti-inflammatory activities to a greater or lesser extent [20]. Flavonoids, which are found commonly in the leaves, flowering tissues and pollens, are an important part of the diet because of their effects on human nutrition [21]. The most important function of these phytochemicals is their antioxidant activity, as they have been shown to be highly effective scavengers of most types of oxidizing molecules, including singlet oxygen and various free radicals [17]. Tepe and Sokmen [22] reported a positive correlation between total phenolic content and their antioxidant activity of *Tanacetum subspecies.* Zhao *et al.* [23] indicated that compounds with reducing power activity are electron donors, and reduce the oxidized intermediates of lipid peroxidation processes, so they can act as primary and secondary antioxidants.

**Table 3:** DPPH free scavenging activities of the leaf part in all varieties of *Labisa pumila.* BHT and α-tocopherol were used as controls.

IC_50_ (µg/mL)		
**Variety**	*Alata*	340.13
	*Pumila*	364.17
	*Lanceolata*	388.29

All analyses were the mean of triplicate measurements.

**Table 4:** Total antioxidant (FRAP) activities of the leaf part in all varieties of *L. pumila* at concentration of 400 μg/mL. BHT, α-tocopherol and Vitamin C were used as positive controls.

Total antioxidant (FRAP) activities (%)		
**Variety**	*Alata*	54.84 ± 0.13 ^c^
	*Pumila*	53.11 ± 0.16 ^d^
	*Lanceolata*	52.17 ± 0.31 ^d^
**Control**	BHT	99.18 ± 0.22 ^a^
	α-tocopherol	96.17 ± 0.19 ^b^
	Vitamin C	99.59 ± 0.11 ^a^

All analyses were mean of triplicate measurements ± standard deviation. Results expressed in percent of free radical inhibition. Means with different letters are significantly different from each other at p < 0.05.

### 2.3. Determination of Phenolics and Flavonoids Compounds by HPLC

In this study, reversed-phase (RP) HPLC was used to identify the flavonoid, isoflavonoid and phenolic compounds in the leaf extracts of all the varieties of *Labisia pumila* Benth. The improvement of extraction efficiency by the microwave method is confirmed by RP-HPLC analysis of the plant extracts. From the obtained results, it is clearly shown that methanolic extracts from leaf part in all varieties exhibited variable patterns of flavonoids, isoflavonoids and phenolics compounds (Table 5 and Table 6). Apigenin, kaempferol, rutin and myricetin were the main flavonoid compounds present in all three varieties, with respective values of 94.72, 217.62, 116.85 and 103.21 µg/g dry sample in the leaves of var. *alata*, 152, 541.78, 51.63, 147.79 µg/g dry sample of var. *pumila*, and 53.92, 157.53, 28.93, 116.68 µg/g dry sample of var. *lanceolata*. Quercitin and the isoflavonoid daidzein were only recorded in var. *pumila* (210 and 142.65 µg/g dry sample, respectively) and *lanceolata* (71.21 and 135.19 µg/g dry sample, respectively). Genistein as another isoflavonoid was only found in var. *lanceolata,* with a value of 107.39 µg/g dry sample. This research also revealed that gallic acid and caffeic acid were the major phenolic compounds in the all extracts, whereas pyrogallol was only observed in *Labisia pumila* var. *alata* (1128.55 µg/g dry sample). The level of kaempferol in the leaves of var. *pumila* was significantly higher than that seen in the *alata* and *lanceolata* varieties*.* These values were lower than the amount of kaempferol found in Chinese tea leaves (1.56–3.31 mg/g dried leaves [24]) but higher than strawberry, with a value 8 µg/g fresh weight [25].

**Table 5:** Concentration of different flavonoids and isoflavonoids in the leaves of three varieties of *Labisia pumila* Benth.

Flavonoid and Isoflavonoid contents (µg/g dry sample)								
Variety	Apigenin	Kaempferol	Myricetin	Naringin	Quercetin	Rutin	Daidzein	Genistein
***Alata***	94.72 ^b^	217.62 ^c^	103.21 ^c^	310.91 ^a^	ND	116.85 ^a^	ND	ND
***Pumila***	152 ^a^	541.78 ^a^	147.79 ^a^	175.14 ^d^	210.01 ^a^	51.63 ^b^	142.65 ^a^	ND
***Lanceolata***	53.92 ^c^	157.53 ^b^	116.68 ^b^	ND	71.21 ^b^	28.93 ^c^	135.19 ^b^	107.39

ND: not detected. All analyses were mean of triplicate measurements ± standard deviation. Results expressed in percent of free radical inhibition. Means with different letters are significantly different from each other at p < 0.05.

**Table 6:** Concentration of different phenolic compounds in the leaves of three varieties of *Labisia pumila* Benth.

Phenolic contents (µg/g dry sample)				
Variety	Gallic acid	Pyrogallol	Caffeic acid	Salicylic acid
***Alata***	623.39 ^a^	1128.55 ^a^	62.13 ^c^	ND
***Pumila***	312.09 ^c^	ND	151.02 ^a^	ND
***Lanceolata***	508.81 ^b^	ND	147.78 ^b^	ND

ND: not detected. All analyses were mean of triplicate measurements ± standard deviation. Results expressed in percent of free radical inhibition. Means with different letters are significantly different from each other at p < 0.05.

Variety *pumila* had demonstrated a significantly higher myricetin level than var. *lanceolata* and *alata*; and they were found to be higher than black current and blueberry, with respective values of 71 and 29 µg/g dry weight [25]. Meanwhile rutin present in all three varieties (*alata*, *pumila* and *lanceolata*) was found to be lower than citrus (3.26 mg/g fresh weight [26] and *Amaranthus viridis* (58.27 µg/mg dry weight) [27]. Furthermore, quercetin present in *L. pumila* var. *pumila* was found to be higher compared to onion (201 µg/g DW) and lower than garlic (227 µg/g dry samples) which was analyzed by Crozier *et al.* [28]. 

**Figure 1:**
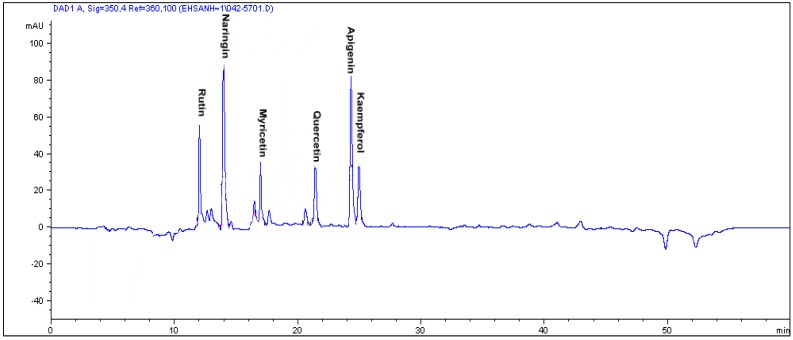
The RP-HPLC chromatogram of flavonoid compounds in the leaves of *Labisia pumila* var. *pumila.* Identification of compounds: rutin, naringin, myricetin, quercetin, apigenin and kaempferol*.*

The results from Table 5 also show that the isoflavonoids levels (daidzein and genistein) in var. *pumila* and var. *lanceolatae* were lower than those found in soybeans with respective values of 341.47 ± 18.96 and 30.03 ± 7.17 mg/kg [29]. As for the phenolic compounds (Table 6), substantial (p < 0.05) amounts of gallic acid were recorded in var. *alata*, followed by var. *lanceolata*; the least came from var. *pumila.* The pyrogallol contents detected in *Labisia pumila* var. *alata* was found to be lower compared to sea grass (*Posidonia aceanica*) with a value of 1.3 mg/g dry weight [30]. Meanwhile, the caffeic acid content in the leaves of all three varieties of *L. pumila*, especially those of var. *pumila* and *lanceolatea*, was found to be higher than that of leaf extract of *Eucalyptus* honey (4.9 mg/g) [31] and apple (8.2 mg/g dry weight) [32]. Secondary metabolites derived from plant such as essential oils, flavonoids and phenolics compounds exhibit biological activities. Compounds such as pyrogallol, gallic acid, naringin and quercetin have been reported to possess antioxidant properties as well as anti-inflammatory activities [33,34]. The HPLC chromatogram in Figure 1 shows the different flavonoids compounds in the leave of *Labisia pumila* var. *pumila* as an instance.

### 2.4. GC-MS Analysis of Three Varieties of Labisia pumila Benth.

Essential oils are volatile and natural complex mixtures of compounds characterized by strong odors and formed by aromatic plants as secondary metabolites [35]. Volatile compounds in essential oils from medicinal and aromatic plants have been known since ancient times to possess many biological activities, especially antibacterial, antifungal and antioxidant properties [36]. The constituents and the percentage values of composition of compounds present in the leaves of *Labisia pumila* var. of *alata*, *pumila* and *lanceolata* are shown in Table 7, Table 8 and Table 9, respectively. The GC-MS analysis of methanolic crude extracts resulted in identification of more than 40 compounds in the leaves of *Labisia pumila* Benth. The obtained results indicated that the main volatile compounds were heptadecanoic acid (20.39%), octadecanoic acid (16.24%) and 2,4,5-trimethyl-1,3-dioxolane (18.69%) in the leaf extracts of *Labisa pumila* var. of *alata*, *pumila* and *lanceolata*, respectively. Leaf of var. *lanceolata* exhibited more volatile compounds (45 compounds) than var. *alata* (31 compounds) and var. *pumila* (24 compounds). The methanolic extracts of leaf from three varieties of *Labisia pumila* contained bioactive compounds that may possess biological properties. Compounds such as acetic acid [37], furfural [38], fumaric acid, dimethyl ester [24], eicosanoic acid and methyl ester [39] have been reported to possess antimicrobial activity. Oleic acid is used as an excipient in pharmaceuticals and as an emulsifying or solubilising agent in aerosol products [40]. The presence of these phytochemicals makes *Labisia pumila* Benth. a potential source of bioactive compounds.

**Table 7:** Chemical composition of methanolic extraction of *L. pumila* var. *alata.*

No.	Composition (%)	Compound
1	2.99	Hydrazine, 1,2-dimethyl
2	0.45	Furfural
3	0.39	*cis*-3-Methyl-2-*n*-propylthiophane
4	0.43	2-Furanmethanol
5	0.45	Benzyl Alcohol
6	0.21	Phenylethyl Alcohol
7	0.19	Ethanone
8	0.48	Octadecanal
9	0.36	2,3 4a, 5,6,7-Hexahydro-1,4-benzodioxin
10	0.32	Menthyl acetate cyclohexanol,5-methyl-2-(1-methylethyl)
11	0.29	Diisobutoxybutane
12	0.36	Cyclododecanol
13	0.25	3-(Hydroxymethyl)-6-(1-methylethyl)-2-cyclohexen-1-one
14	2.31	Hexadecanoic acid methyl ester
15	9.14	2,4,5-Trimethyl-1,3-dioxolane,
16	0.30	Benzene-1,2,3,4-tetraol
17	0.42	N-Ethyl-N-nitrosoethanamine,
18	0.25	Isobenzofuran
19	3.72	10-Octadecenoic acid methyl ester
20	0.59	Ethyl oleate
21	9.36	9,12-Octadecadienoic acid methyl ester
22	0.51	2-Propylthiophene,
23	1.79	Linoleic acid ethyl ester
24	7.76	11,14,17-Eicosatrienoic acid
25	1.61	9,12,15-Octadecatriene
26	1.07	Phytol
27	20.39	Heptadecanoic acid
28	1.71	Octadecanoic acid
29	6.72	9-Hexadecenoic acid
30	16.15	9,12-Octadecadienoic acid
31	9.03	9,12, 15-Octadecatriene

**Table 8:** Chemical composition of methanolic extraction of *L. pumila* var. *pumila.*

No.	Composition (%)	Compound
1	0.31	Guanidine
2	2.73	Methyl formate
3	0.31	Methoxypyrazine
4	0.33	2-Furanmethanol
5	1.22	Benzyl alcohol
6	0.49	Tetradecyloxirane
7	0.45	3,7,11,15-Tetramethyl-2-hexadecen-1-ol
8	0.54	Eicosenoic acid methyl ester
9	2.36	Hexadecanoic acid
10	12.68	2,4,5-Trimethyl-1,3-dioxolane,
11	0.39	5-Hydroxy-2-methylthiopyrimidine
12	0.66	2,3-Dihydrobenzofuran
13	3.75	10-Octadecenoic acid methyl ester
14	11.18	9,12-Octadecadienoic acid methyl ester
15	11.07	9,12,15-octadecatriene
16	0.44	2,4-Dimethylphenol
17	0.96	Phytol
18	0.51	D-Tyrosine
19	0.87	Anthracene
20	16.24	Octadecanoic acid
21	0.65	*n*-Hexadecanoic acid
22	5.97	9 Hexadecenoic acid
23	13.40	9,12-Octadecadienoic acid
24	12.47	9,12,15-Octadecatrien-1- ol , (Z,Z,Z)

**Table 9:** Chemical composition of methanolic extraction of *L. pumila* var. *lanceolata*.

No.	Composition (%)	Compound
1	2.27	Methyl formate
2	0.34	Propanedioic acid
3	0.29	*Z*-β-Terpineol
4	0.70	Furanone
5	1.37	Hexanoic acid
6	3.79	Benzyl alcohol
7	0.34	Phenylethyl alcohol
8	0.93	Hexanoic acid
9	1.54	Tetradecyloxirane
10	0.62	Triethylenediamine
11	0.48	Dodecyloxirane,
12	0.46	2,5-Dimethyl-4-hydroxy-3(2H)-furanone
13	0.38	Dihydro-3-hydroxy-4,4-dimethyl-2(3*H*)-furanone
14	1.18	Cyclododecanol
15	0.78	1,6,10-Dodecatrien-3-ol
16	0.54	3-Methylphenol,
17	2.88	Pentadecanoic acid
18	18.69	2,4,5-Trimethyl-1,3-dioxolane
19	2	D-Gluconic acid
20	1.53	Benzene-1,2,3,4-tetraol
21	0.44	2,5-bis(1,1-Dimethylethyl)phenol,
22	0.70	1-Methoxy-9-octadecene
23	0.36	8-Methoxy-1,6-octadiene
24	0.89	2,3-Dihydrobenzofuran
25	0.71	Benzoic acid
26	1.95	9-Octadecenoic acid
27	0.34	Lauric anhydride
28	3.87	11,14-Ecosadienic acid
29	0.95	Cyclopropaneoctanoic acid
30	3.29	9,12,15 Octadecatrien-1-ol
31	0.39	3-*tert-*Butyl-4-hydroxyanisole
32	4.21	Phytol
33	0.55	Heptadecanoic acid
34	1.23	D-Tyrosine
35	11.96	*n*-Hexadecanoic acid
36	0.48	Undecanentrile
37	0.48	4-Hydroxy-3,5-dimethoxybenzoic acid ,
38	0.50	2,6,10,14,18-Pentamethyl-2,6,10,14,18-eicosapentaene
39	1.02	Octadecanoic acid
40	5.45	9-Hexadecenoic acid
41	1.57	Di-*n*-octyl phthalate (DNOP)
42	7.37	9,12-Octadecadienoic acid
43	5.36	11,14,17-Ecosatrienoic acid
44	1.65	2-(But-2-enylideneamino)-propionitrile
45	3.14	N-aminoacetyltyramine,

## 3. Experimental

### 3.1. Plant Material

Seedlings of *Labisia pumila* varieties *alata*, *pumila*, and *lanceolata* were, respectively, collected from their places of origin at Hulu Langat, Selangor; Sungkai, Perak; and Kota Tinggi, Johore, and raised under glasshouse for 18 months before being used in the study. Healthy and uniform seedlings in term of leaf numbers were selected from the three varieties. The leaves of three varieties of *Labisa pumila* Benth. were cleaned, separated, and freeze dried for further analysis.

### 3.2. Microwave Assisted Extraction (MAE)

MAE was performed on microwave apparatus using closed vessel system with pressure (ETHOS^®^ T Microwave digestion/extraction system, Milestone Co., Italy) based on the method described by Xiao *et al.* [41] with some modification. One gram of leaf part of three varieties of *Labisia pumila* was weighed using a clean aluminum container, then transferred into vessel of the Ethos E Microwave Extraction System and extracted with methanol (30 mL) for 2 min (p = 750 w). The extraction temperature was applied to 60 °C. After extraction, the vessels were allowed to cool at room temperature before opening. Then the extracts were filtered and stored in the refrigerator.

### 3.3. Total Phenolics Determination

Total phenolics content was determined by using Folin–Ciocalteu reagent according to Ismail *et al.* [42] and total phenolic results were expressed as mg gallic acid equivalents/g dry matter of the plant material. 

### 3.4. Total Flavonoids Determination

Total flavonoids were determined based on aluminium chloride colorimetric assay described by Ismail *et al.* [42]. Total flavonoid compound of extracts were expressed as mg rutin equivalent/g dry matter of the plant material.

### 3.5. Analyses of Phenolic and Flavonoid Compounds by RP-HPLC

The phenolic and flavonoid compounds of the leaf of three varieties of *Labisia pumila* Benth. were quantitatively measured by reversed-phase HPLC based on the method described by Crozier *et al.* [28] with some modification. Phenolic standards were gallic acid, salicylic acid, caffeic acid and pyrogallol. Flavonoid standards were quercetin, rutin, myricetin, kaempferol, naringin, apigenin and isoflavonoid standards were genistein and daidzein. An aliquot of sample extracts was loaded on the HPLC equipped with an analytical column Intersil ODS-3 (5 μm 4.6 × 150 mm, Gl Science Inc). Solvents comprising deionized water (solvent A) and acetonitrile (solvent B) were used. The PH of water was adjusted to 2.5 with trifluoroacetic acid. The phenolic and isoflavonoid compounds were detected at 280 nm while flavonoid compounds at 350 nm. The column was equilibrated with 85% solvent A and 15% solvent B. Then the ratio of solvent B was increased to 85% in 50 min followed by reducing solvent B to 15% in 55 min. This ratio was maintained to 60 min for the next analysis with flow rate at 0.6 mL/min.

### 3.6. Antioxidant Activity

#### 3.6.1. DPPH Radical-Scavenging Activity

Free radical scavenging activity of extracts were determined with 1,1-diphenyl-2-picryl-hydrazil (DPPH) as free radicals according to Ismail *et al.* [42]. The absorbance was measured at 515 nm by using a spectrophotometer. Butylated hydroxytoluene (BHT) and α-tocopherol were used as standard antioxidants.

#### 3.6.2. Ferric-Reducing Antioxidant Power (FRAP) Assay

The ferric reducing antioxidant power (FRAP) of the extracts was determined as described by Yen and Chen [43]. Ascorbic acid, BHT and α-tocopherol were used as standard antioxidants. 

### 3.7. Gas Chromatography-Mass Spectrophotometry Method (GC-MS)

The GC-MS analysis of methanolic crude extract of the leaf of three varieties of *Labisia pumila* were quantitatively performed by GC-MS (Shimadzu QP2010PLUS system) equiped with a capillary column (30 m × 0.25 mm i.d. × 0.25 μm film thickness) based on the method described by Hossain and Rahman [44] with some modification. Split less injection was performed with a purge time of 1.0 min. The carrier gas was helium at a flow rate of 1 mL min^−1^. The column temperature was maintained at 50 °C for 3 minutes, then programmed at 5 °C min^−1^ to 80 °C and then at 10 °C min^−1^ to 340 °C. The inlet temperature was 250 °C, the detector temperature was 340 °C and the solvent delay was 4 min. The identification of the peaks was based on computer matching of the mass spectra with the National Institute of Standards and Technology (NlST 08 and NIST 08s) library and by direct comparison with published data.

### 3.8. Statistical Analysis

The antioxidant activities, total flavonoids, total phenolics contents and profiling of phenolics and flavonoids compounds were analyzed using analysis of variance (ANOVA) with Statistical Analysis System (SAS) Version 9 (SAS Institute, Cary, NC, USA). Significant differences among means from triplicate analyses (p < 0.05) were determined by Duncan’s Multiple Range Test [45]. 

## 4. Conclusions

Microwave extraction method was used to extract the flavonoids, isoflavonoids and phenolics compound from the leaves of three varieties of *Labisia pumila* Benth. and their antioxidant properties determined. Varietal compounds and compositions were also screened using GC-MS.

The results demonstrate that *L. pumila* extracts contain variable patterns of flavonoids, phenolic and various bioactive volatile compounds. These compounds possess noticeable antioxidant activity. Plant secondary metabolites are far more restricted than plant primary metabolites and often accumulated in small quantities [46]. They are known as flavonoids, phenolics compounds, essential oils, curcuminoids and others. They posses anti-oxidant, anti-inflammatory, anti-aging and a lot more functions [47]. The overall result obtained from this research suggested that all varieties of *Labisia pumila* Benth. are a source of bioactive compounds endowed with interesting antioxidant activities. Thus, the presence of phytochemicals and other bioactive compounds present in this plant may serve as a new potential source of medicines in the future. 
